# Validation of Novaprep^®^ HQ+ liquid-based cytology medium for high-risk human papillomavirus detection by hc2

**DOI:** 10.1186/s13027-016-0092-7

**Published:** 2016-08-17

**Authors:** David Guenat, Sophie Launay, Didier Riethmuller, Christiane Mougin, Jean-Luc Prétet

**Affiliations:** 1 COMUE UBFC, Univ Franche-Comte, F-25000 Besancon, France; 2 CHRU Besancon, F-25000 Besancon, France; 3 EA 3181, LabEx LipSTIC ANR-11-LABX-0021, FED4234, F-25000 Besancon, France; 4 Inserm CIC 1431, F-25000 Besancon, France; 5 Laboratoire de Biologie Cellulaire et Moléculaire, Centre Hospitalier Régional Universitaire, Boulevard A Fleming, 25030 Besançon cedex, France

**Keywords:** Papillomavirus, Liquid based cytology, HPV testing, Validation, Hybrid capture 2

## Abstract

**Background:**

Preanalytical conditions determine the reliability and validity of bioassays. Therefore, the analytic performances of biological tests need to be determined when preanalytical steps differ from those recommended by the manufacturer. The objective of the study was to assess the analytic performance of the hc2 test for the detection of high-risk HPV DNA from cells stored in the new Novaprep® HQ+ medium.

**Methods:**

Repeatability, reproducibility, method comparison and stability (-20 °C, +4 °C, +20 °C and +40 °C up to six months) were evaluated from HPV16 and HPV18 positive cell lines diluted in the Novaprep® HQ+ medium and the reference Specimen Transport Medium (STM). A series of cervical samples with atypical squamous cells of undetermined significance (ASC-US) cytology and stored in the Novaprep® HQ+ medium was also tested.

**Results:**

Coefficients of variation for repeatability and reproducibility were less than 8 %. Method comparison showed perfect agreement in hc2 results when the HPV-positive cells were diluted in HQ+ and reference media. Stability experiments demonstrated that the storage conditions did not alter the hc2 test results. Furthermore, clinical samples were adequately preserved for hc2 testing.

**Conclusions:**

Overall, our data show that the new Novaprep HQ+ medium is suitable for high-risk HPV testing by hc2.

## Background

According to Arbyn’s meta-analysis, the sensitivity of both conventional and liquid-based cytology (LBC) remains low (below 60 %) for the detection of high grade squamous intraepithelial lesions (HSIL) of the cervix [1]. High-risk human papillomavirus (hrHPV) testing has been proposed to overcome the limits of cervical cytology for the triage of women with atypical squamous cells of undetermined significance (ASC-US) cytology [2]. Moreover, alone or in combination with cytology, hrHPV testing allows screening of HSIL or cancers with high sensitivity and negative predictive value [3–6]. In this way, introducing HPV testing as a primary screening test may improve the safety and cost-effectiveness of cervical cancer screening [4, 7–10].

Among the most reliable HPV DNA tests, the hybrid capture 2 test (hc2) is a nucleic acid hybridization assay with signal amplification. This technique has been validated by the manufacturer using the Specimen Transport Medium (STM) (Qiagen, Hilden, Germany), the ThinPrep Pap Test (Hologic, Marlborough, MA, USA) and the BD SurePath™ liquid-based Pap test (BD, Franklin Lakes, NJ, USA) to collect cervical cells. In 2010, we conducted a clinical study to assess the suitability of the LBC Novaprep® Vial Test system (Novacyt, Vélizy-Villacoublay, France) for hrHPV DNA detection in cervical samples using the hc2 test. Very high agreement for hrHPV DNA detection was found between two consecutive cervical samples deposited in the Novaprep® Vial Test and in the reference medium STM (Qiagen) [11]. Since this study, the fixative composition of the Novaprep® system has been slightly modified and the product is now called Novaprep® HQ+ Orange. The objective of this study was to assess whether the modifications of the Novaprep LBC medium impact the performance of the hc2 assay to detect hrHPV DNA. A comprehensive evaluation of repeatability, reproducibility, method comparison and stability was conducted to identify potential sources of variation and interference.

## Methods

### Cell lines

The human Ca Ski (HVP16, 400 to 600 copies per cell) and SiHa (HVP16, 1 to 2 copies per cell) cell lines, the HeLa (HPV18, 20 to 40 copies per cell) cell line and the HPV negative C33-A cell line were obtained from ATCC (Manassas, VA, USA) and routinely tested for the presence of HPV in our laboratory. Ca Ski and SiHa cells were maintained at 37 °C (5 % CO_2_) in complete RPMI or DMEM respectively (Lonza, Levallois-Perret, France) supplemented with 10 % fetal bovine serum (FBS; Lonza), 5x10^4^ U/L penicillin/streptomycin (Lonza) and 2 mM L-glutamine (Lonza). HeLa and C33-A cells were grown in complete EMEM (Lonza) supplemented with 10 % FBS, 5x10^4^ U/L penicillin/streptomycin and 2 mM L-glutamine.

HPV positive and HPV negative cells were diluted at indicated concentrations in the STM (Digene/Qiagen, Courtaboeuf, France) as reference medium and in the Novaprep® HQ+ Orange medium (Novacyt, Vélizy-Villacoublay, France), an alcohol-based fixative primarily dedicated to liquid-based cytology. Because pretreatment of viscous samples with dithiothreitol (DTT), a mucolytic agent, may be recommended to provide mucus-free single-cell suspensions, Novacyt also provided the Novaprep® HQ+ orange containing 0.03 % DTT. For convenience, the Novaprep® HQ+ medium without DTT will be referred to as HQ + A and the Novaprep® HQ+ medium with DTT will be referred to as HQ + B.

### Cervical samples

Three hundred and twenty two consecutive samples collected at the endocervical-ectocervical junction with a cytobrush, conserved in Novaprep® HQ+ Orange medium and with an ASC-US diagnosis were routinely tested for hrHPV with the Digene hc2 High-Risk HPV DNA Test (Qiagen). All samples were stored in a biobank approved by the local ethics committee (Comité de Protection des Personnes Est II), and for which a declaration of collection and storage of human samples for research use has been lodged with the French Ministry of Higher Education and Research (Ministère de l’Enseignement Supérieur et de la Recherche) (declaration number DC-2014-2086).

### Repeatability and reproducibility

Repeatability of hc2 testing was assessed by calculating the mean, the standard deviation (SD), and the coefficient of variation (CV) of relative light units/cutoff (RLU/CO) from 14 replicates prepared extemporaneously with medium HQ + A and medium HQ + B. This assessment was conducted using three concentration levels of HPV16 + cells (corresponding to 1.25x10^5^ and 1.25x10^6^ SiHa cells and 6.25x10^4^ Ca Ski cells per mL) and HPV18 + cells (corresponding to 3.12x10^4^, 6.25x10^4^ and 6.25x10^5^ HeLa cells per mL) expected to give clinically relevant RLU/CO values. Assessment of the reproducibility of hc2 testing was conducted using three different batches of HQ + A and HQ + B media (namely HQ+ A1; HQ+ A2; HQ+ A3; HQ+ B1; HQ+ B2; HQ+ B3). Fourteen replicates of 1.25x10^5^ SiHa cells/mL were prepared extemporaneously with the different batches of media. The mean, SD and CV of RLU/CO were calculated from 42 replicates for each media.

### Method comparison

Method comparison was conducted using six HPV16 positive samples and 6 HPV negative samples prepared extemporaneously with the reference medium STM and with HQ + A and HQ + B media. HPV positive samples consisted of 5x10^4^ and 2.5x10^4^ Ca Ski cells/mL and 2.5x10^6^, 1.25x10^6^, 6x10^5^ and 3x10^5^ SiHa cells/mL. The HPV negative C33-A cells were used to obtain 2.5x10^6^, 1.25x10^6^ and 6x10^5^, 3x10^5^, 1.5x10^5^ and 0 cells/mL. Each sample was analyzed in duplicate. The sensitivity and specificity of hrHPV detection were calculated for each medium.

### Stability

For stability, the effect of time and temperature of storage was analyzed using freshly prepared SiHa cells (1.7x10^5^ cells/mL) stored in the three batches of HQ + A and HQ + B media up to 6 months at 18 to 25 °C (hc2 analysis was performed at weeks 0, 2, 4, 6, 8, 10, 12, 16, 24 and 28) and at -15 to -30 °C (hc2 analysis at weeks 2, 4 and 28) and up to 3 months at 2 to 8 and 40 °C (hc2 analysis at weeks 2, 4, 6, 8, 10, 12 and 16).

### Human papillomavirus DNA detection

High-Risk HPV DNA testing was performed with the hc2 High-Risk HPV DNA Test® (Qiagen/Digene) according to the manufacturer’s instructions. For the denaturation step, the protocol dedicated to samples collected in the PreservCyt solution was applied to cellular samples (cell lines or cervical samples) collected in Novaprep® HQ+ Orange media without and with DTT. Briefly, 0.1 volume of sample conversion buffer (Qiagen) was added to each tube containing 1 mL of HPV positive or HPV negative cells collected and stored in Novaprep®HQ+ orange. After vortexing, cells were centrifuged at 2900 *g* for 15 min (±2 min) and supernatant was carefully decanted. Then 300 μL (1 mL for the method comparison) of Specimen Transport Medium/Denaturing Reagent mixture (in a 2:1 ratio) was added to cell pellets that were resuspended by vortexing. The denaturation step was conducted for 15 min at 65 °C, then specimens were vortexed and incubated at 65 °C for another 30 min. A 75 μL aliquot of denatured samples was processed for hybridization as recommended by the manufacturer. All experiments were conducted with the same batch of hc2 High-Risk HPV DNA Test®.

## Results

Means, SD and CV for repeatability and reproducibility are presented in Tables 1 and 2 respectively. The CV for repeatability varied from 2.4 to 7.6 % according to the media tested and the genotype detected. As regards reproducibility, the CV were in the same range as those obtained for repeatability, namely 6 % for the three batches of medium HQ + A and 8 % for the three batches of medium HQ + B. As a whole, these CV were very satisfactory and were lower than the CV proposed by the manufacturer for their internal Quality Control (25 %).

**Table 1:** Repeatability analysis of HPV16 and HPV18 DNA by hc2

	Mean RLU/CO	SD	CV%
HPV16 HQ+ A			
Low	32	1.4	4.3
Medium	231	10.5	4.5
High	2780	69.9	2.5
HPV16 HQ+ B			
Low	35	1.3	3.7
Medium	247	12.5	5.1
High	2953	69.6	2.4
HPV18 HQ+ A			
Low	9	0.7	7.6
Medium	18	0.8	4.1
High	146	7.0	4.8
HPV18 HQ+ B			
Low	11	0.5	4.6
Medium	21	1.1	5.5
High	158	8.4	5.3

*RLU/CO* relative light units/cutoff, *SD* standard deviation, *CV* coefficient of variation, *HQ+ A* Novaprep® HQ+ medium without DTT, *HQ + B* Novaprep® HQ+ medium with DTT

**Table 2:** Reproducibility analysis of HPV16 DNA by hc2 using 3 batches of medium A and B

Batches	HQ+ A1	HQ+ A2	HQ+ A3	HQ+ B1	HQ+ B2	HQ+ B3
Mean (each series)	27	27	29	30	29	29
Mean all series		28			29	
SD all series		1.7			2.4	
CV%		6			8	

Abbreviations as in Table 1

For method comparison, the ability of the hc2 test to assign the sample status without error was assessed from HPV16 positive and HPV negative cells diluted in HQ + A and HQ + B media in comparison with cells diluted in the STM reference medium. As shown in Table 3, all HPV16 positive samples gave a RLU/CO > 1 and all HPV negative samples gave a RLU/CO < 1, regardless of the medium. Accordingly, the sensitivity and specificity of the hc2 test for hrHPV DNA detection from cells diluted in HQ + A and HQ + B media were 100 %, as was the case for cells diluted in STM. Furthermore, the non-parametric Friedman test showed that the RLU/CO values given by HPV positive samples did not differ according to the medium used to collect cells.

**Table 3:** Method comparison

		hc2	
	Pos	Neg	Total
STM pos	12	0	12
STM neg	0	12	12
total	12	12	
HQ+ A pos	12	0	12
HQ+ A neg^a^	0	11	11
total	12	11	
HQ+ B pos	12	0	12
HQ+ B neg	0	12	12
total	12	12	
		Se	Sp
STM		100	100
HQ+ A		100	100
HQ+ B		100	100

*Pos* positive, *Neg* negative

^a^One missing sample due to error in distribution

*Se* sensitivity, *Sp* specificity

*HQ+ A* Novaprep® HQ+ medium without DTT, *HQ + B* Novaprep® HQ+ medium with DTT

hrHPV DNA stability was tested at four different temperatures, two of which corresponded to worst-case conditions (between -15 to -30 and +40 °C). As shown in Table 4, the RLU/CO values given by the hc2 test were very similar over the entire duration of the experiment, irrespective of the medium, batch and storage temperature. We observed slightly lower RLU/CO values for cells collected in the medium with DTT and stored at 4 °C than for other conditions.

**Table 4:** Stability of samples diluted in media A and B

	RLU/CO																							
	18 °C to 25 °C						2 °C to 8 °C						40 °C						-15 °C to -30 °C					
Weeks	A1	A2	A3	B1	B2	B3	A1	A2	A3	B1	B2	B3	A1	A2	A3	B1	B2	B3	A1	A2	A3	B1	B2	B3
0	41	42	36	37	37	39	-	-	-	-	-	-	-	-	-	-	-	-	-	-	-	-	-	-
2	51	48	45	36	47	62	49	52	48	36	41	41	51	67	51	48	59	48	52	46	46	48	50	48
4	51	42	47	44	46	53	54	49	44	36	45	43	53	54	50	49	39	43	47	45	48	49	57	50
6	49	51	46	46	46	50	53	52	52	31	35	35	47	44	52	45	49	45	-	-	-	-	-	-
8	51	54	50	41	50	43	48	55	40	35	38	32	54	49	55	40	46	52	-	-	-	-	-	-
10	41	41	42	38	36	38	41	45	39	28	36	33	42	40	42	40	42	38	-	-	-	-	-	-
12	45	42	43	35	40	47	49	40	46	29	33	29	50	47	47	37	43	42	-	-	-	-	-	-
16	48	52	50	42	43	33	49	51	47	34	31	36	49	48	42	44	47	42	-	-	-	-	-	-
24	34	33	33	33	29	31	-	-	-	-	-	-	-	-	-	-	-	-	-	-	-	-	-	-
28	44	43	42	43	39	36	-	-	-	-	-	-	-	-	-	-	-		43	45	42	40	40	43

*RLU/CO* relative light units/cutoff

Among the 322 cytology samples from women with atypical squamous cells of undetermined significance (ASC-US), 204 were hrHPV positive and 118 were hrHPV negative.

## Discussion

Accreditation of medical laboratories according to ISO 15189 standards is becoming a requirement for medical laboratories throughout Europe [12]. This implies that laboratory procedures, which include the preanalytical steps, must be validated and verified before applied for clinical testing, in order to ensure reliable results. Indeed, evaluations of biological tests, independently from the manufacturers, are crucial to choose the most appropriate test [13].

According to the manufacturer, the hc2 test has CE In Vitro Diagnostics (IVD) certification for cell samples harvested in 3 media, namely STM (Qiagen), PreservCyt (Hologic) and SurePath (BD). Some studies have reported the potential use of cell samples processed with different liquid-based cytology media for hrHPV testing by hc2 [11, 14–17]. We report here that the performance characteristics of the hc2 assay are appropriate for its intended use with cells stored in the new Novaprep® HQ+ medium with or without DTT. Thus, pretreatment of cervical smears with a mucolytic agent (DTT up to 0.03 %) to obtain single-cell suspensions does not alter the specimen for further hrHPV DNA testing by hc2. In the same way, the adjunct of glacial acetic acid (GAA) in LBC specimen to lyse red blood cells has been evaluated and studies confirmed that GAA pretreatment had no significant impact on analytical and clinical performance of HPV tests [18–20].

To identify potential sources of variation and/or interference due only to fixatives, each series of experiments (repeatability, reproducibility, method comparison and stability) was conducted using unique batches of cells and hc2 kits from the same batch number.

The coefficients of variation of the hc2 test ranged from 2.4 to 7.6 % for repeatability and 6 to 8 % for reproducibility. Such CVs are very satisfactory. Indeed, they are lower than 25 %, the maximum CV value that may be allowed within each series of samples to validate the test in quality controls. Moreover, the CVs observed here are lower than the value obtained with our own internal quality control, namely 18 %.

The method comparison made it possible to assess the robustness of hrHPV DNA detection by hc2 with cell samples prepared with Novaprep® HQ+ orange with or without DTT, in comparison with cells samples prepared with STM. Again, the results were more than adequate. hrHPV detection was as efficient with cells diluted in the two Novaprep® HQ+ orange media (A and B) as with cells diluted in STM. Furthermore, no cell samples stored in Novaprep® HQ+ orange medium and known to be positive or negative for the target were misclassified (false positive or false negative) after hrHPV DNA testing with the hc2 test.

Ensuring the stability of cervical samples is mandatory, since HPV testing may be delayed. Indeed, screening algorithms recommend hrHPV DNA testing as a triage test for women with cytological abnormalities. This is the case in France, where reflex HPV is an option following ASC-US cytology. Furthermore, in some situations, samples must be shipped to a distant molecular biology laboratory in charge of hrHPV testing. The stability experiment conducted in this study demonstrates that both HQ + A and HQ + B media adequately preserve hrHPV DNA and allow for its efficient detection with hc2 up to 6 months at room temperature (18-25 °C), and up to 3 months in refrigerated conditions (+4 °C). Moreover, excellent stability was demonstrated in the two “worst-case” scenarios, namely samples stored betwen -15°C to -30°C for 6 months and at +40 °C for 3 months. The Novaprep® HQ+ orange media with or without DTT are particularly well adapted for countries with a tropical or equatorial climate, where air-conditioning may not be systematically available. In a practical point of view, the rate of hrHPV detection from cervical samples with ASC-US cytology stored in the Novaprep® HQ+ LBC medium was 63 %. This rate, similar to that published with ASC-US samples stored in SurePath media [21], indicates a good hrHPV DNA preservation.

The robustness of the Novaprep HQ+ medium for hrHPV DNA detection was also recently demonstrated with the real-time PCR based Cobas® 4800 HPV test [22]. Although the study by Khiri, as well as our own results, suggest that it would be possible to use cells collected in Novaprep® HQ+ orange medium for hrHPV DNA detection by molecular testing, this does not by any means exempt each individual laboratory from the need to verify/validate the method according to ISO15189 standards before using this medium in routine practice. Furthermore, Hortlund et al. suggested that the clinical sensitivity of HPV test should be annually assessed to warrant high quality results [23].

## Conclusion

This study provides evidence that the Novaprep® HQ+ orange medium does not alter viral DNA preservation and does not render cell samples inadequate for hrHPV DNA testing with hc2.

## Abbreviations

ASC-US, atypical squamous cells of undetermined significance; CV, coefficient of variation; DMEM, Dulbecco’s Modified Eagle Medium; DTT, dithiothreitol; EMEM, Eagle’s Minimal Essential Medium; FBS: Fetal Bovine Serum; GAA, glacial acetic acid; hc2, hybrid capture 2; HPV, human papillomavirus; hrHPV, high-risk human papillomavirus; HSIL, high grade squamous intraepithelial lesion; LBC, liquid-based cytology; RLU/CO, relative light units/cutoff; RPMI, Roswell Park Memorial Institute medium; SD, standard deviation; STM, specimen transport medium
